# Rapid and simultaneous purification of aflatoxin B1, zearalenone and deoxynivalenol using their monoclonal antibodies and magnetic nanoparticles

**DOI:** 10.1007/s43188-020-00083-w

**Published:** 2021-01-28

**Authors:** Hyuk-Mi Lee, Hwan-Goo Kang

**Affiliations:** 1 Animal and Plant Quarantine Agency, 177 Hyeoksin 8-ro, Gimcheon, Gyeongsangbuk-do 39660 Republic of Korea; 2 Present Address: College of Biohealth, Semyung University, Semyungro 65, Jecheon, Chungcheongbuk-do 27136 Republic of Korea

**Keywords:** Mycotoxins, Antibody, Magnetic nanoparticle, Purification, Feed

## Abstract

To develop a new simple and simultaneous purification method for mycotoxins in feeds and grains, magnetic nanoparticles (MNPs) conjugated with monoclonal antibodies (mAbs) against mycotoxins were used to separate aflatoxin B1 (AFB_1_), zearalenone (ZEA) and deoxynivalenol (DON). For a single spike of each mycotoxin into the buffer solution (16% MeOH in PBS), mean recoveries were 93.1–95.0% for AFB_1_ (5–20 ng/mL spiked), 87.2–96.0% for ZEA (125–500 ng/mL spiked) and 75.2–96.9% for DON (250–1,000 ng/mL spiked) by HPLC and ELISA. Recoveries of AFB_1_ (20 ng/mL) and ZEA (500 ng/mL) simultaneously spiked into the buffer solution were 87.0 and 99.8%, respectively. Recovery rates of AFB_1_/DON and DON/ZEA spiked simultaneously were 86.2%/76.6% and 92.0%/86.7%, respectively, at concentrations of 20 ng/mL AFB_1_, 500 ng/mL ZEA, and 1,000 ng/mL DON. Recoveries using the novel mAb–MNP conjugated system in a buffer solution simultaneously spiked with AFB_1_, ZEA and DON were 82.5, 94.6 and 73.4%, respectively. Recoveries of DON in animal feed were 107.7–132.5% at concentrations of 250–1,000 ng/g spiked in feed. The immunoaffinity chromatography (IAC) clean-up method was compared with the purification method using novel mAb–MNP. After fortification of animal feed with AFB_1_ (5, 10 and 20 ng/g feed) and ZEA (125, 250 and 500 ng/g feed), AFB_1_ and ZEA were purified using both the methods. In the case of the novel mAb-MNP conjugated system, mean recoveries for AFB_1_ were 89.4, 73.1 and 88.3% at concentrations of 5, 10 and 20 ng/g feed, respectively. For ZEA, mean recoveries were 86.7, 85.9 and 79.1% at concentrations of 125, 250 and 500 ng/g, respectively. For IAC purification, recoveries were 42.9–45.1% for AFB_1_ and 96.8–103.2% for ZEA. In conclusion, the present purification method using monoclonal antibodies conjugated to MNPs can be used for simple and simultaneous purification of mycotoxins from feed and maize.

**Supplementary Information:**

The online version contains supplementary material available at 10.1007/s43188-020-00083-w.

## Introduction

Mycotoxins produced by fungi in grains and animal feeds threaten animal and human health. Among these, aflatoxin B_1_ (AFB_1_), zearalenone (ZEA) and deoxynivalenol (DON) are commonly found in animal feeds and grains. Detection of mycotoxins is therefore important for preventing animals and humans from consuming feed or food contaminated with these toxins. High-performance liquid chromatography (HPLC) [1], and HPLC—tandem mass spectrometric methods [2] have been used to quantitatively determine toxin concentrations in grains and biological samples, However, these methods require expensive, time-consuming extraction steps, which require use of hazardous organic solvents. To replace these steps, immunoaffinity chromatography (IAC) combined with antibodies has become a popular method for isolating mycotoxins from samples [3, 4]. However, IAC can also be expensive and time-consuming. Recently, magnetic microbeads and nanoparticles combined with antibodies have drawn attention as novel tools for the isolation of chemicals from grains and biological samples [5, 6]. Magnetic separation has also been suggested as a novel tool for isolating bacteria from ground beef [7]. The combination of magnetic separation and real-time polymerase chain reaction (PCR) can achieve rapid and sensitive quantitative detection of microorganisms without the requirement of an enrichment culture step [8, 9]. Compared with microbead-based immunomagnetic separation, magnetic nanoparticles (MNPs) enhance capture efficiency by removing the requirement of vigorous mechanical mixing during separation. In our previous study, MNP was successfully applied for isolating DON from animal feed using its specific monoclonal antibody (mAb) [10]. Few studies have been conducted to simultaneously separate mycotoxins using MNP. The present study aimed to develop an advanced multi-purification tool for three mycotoxins in animal feed and grains using mAbs for each mycotoxin and MNPs to facilitate purification by magnetism.

## Materials and methods

### Chemicals and reagents

Standards of mycotoxins (AFB_1_, ZEA and DON), carbonate–bicarbonate buffer glutaraldehyde solution (Grade II, 25%), glycine, tris (hydroxymethyl) amino-methane (ACS reagent, 99.8 + %) and sodium chloride (ACS reagent,  ≥ 99.0%) were purchased from Sigma–Aldrich (St. Louis, Missouri, USA). Skim milk (BD, Difco™ skim milk, Sparks, NV, USA), Tween 20 (molecular biology grade, Applichem, Darmstadt, Germany), SureBlue™ TMB Microwell peroxidase substrate (1-component) (KPL, Gaithersburg, MA, USA), sulfuric acid (Applichem, 95%–98% pure NF grade, Darmstadt, Germany), pyridine (Wako, Osaka, Japan), methanol (MeOH) (Merck, Darmstadt, Germany) and bovine serum albumin (BSA) (Fluka, St. Louis, USA) were purchased from the mentioned companies. The Micro BCA™ Protein Assay Kit (Thermo Scientific, Rockford, IL, USA) and commercial ELISA Kit for AFB_1_, ZEA and DON 2/3 (8335) (NEOGEN, Lansing, MI, USA) were used for protein and mycotoxin determination. Immunoaffinity columns for AFB_1_ (NEOGEN, Glasgow, UK) and ZEA (R-BIOPHARM RHÔNE LTD, Glasgow, Scotland) were used to purify mycotoxin from a liquid solution. HPLC-grade acetonitrile (ACN), MeOH and water were purchased from J. T. Baker Inc. (Phillipsburg, NJ, USA). Phosphate-buffered saline (PBS) buffer was purchased from Biosesang Inc. (Seongnam, Republic of Korea).

### mAbs and MNPs

The following mAbs were produced in our laboratory: kj-AFB against AFB_1_, kk-ZEA against ZEA [11] and NVRQS-DON against DON [10]. Amine-functionalised MNPs (SPM-NH_2_) used in the present study were produced at Nanobirck (Suwon, Republic of Korea). In a 1,000 mL three neck flask, 500 mL of MeOH and 250 mL of 3-aminopropyltriethoxysilane (APTES) were mixed. Superparamagnetic nanoparticles (SPMs) were dispersed in distilled water (DW) by sonication and then these homogeneous SPMs were injected into the mixed solution. The solution was heated at 60 °C for 3 h with stirring and allowed it to be cooled to room temperature. These amine-functionalized MNPs were washed three times with ethanol (EtOH) and finally dispersed in DW.

### Determination of mycotoxins

AFB_1_ and ZEA were quantified by HPLC. The Waters HPLC System (Waters, Milford, MA, USA) consisted of a 2695 separation module, photodiode array detector 2996 and multi λ fluorescence detector 2475 and was controlled with Waters Empower software. AFB_1_ was analyzed by fluorescence detector with the excitation and emission wavelengths set at 365 and 435 nm, respectively. Also, ZEA was detected by fluorescence with excitation wavelength set at 274 nm and emission wavelength at 440 nm. Quantification of AFB_1_ and ZEA was performed by measuring peak areas at their retention time (10–11 min for AFB_1_ and 5–5.5 min for ZEA) and the peak area of the samples was compared with the peak area of standards of mycotoxins to calculate concentration. AFB_1_ separations were performed using Waters XTerra^®^ RP18, with dimensions of 250 4.6 mm I.D and 5 μm particle size. The mobile phase was ACN/MeOH/water (1:1:3, v/v); the flow rate was 1.0 mL/min and the column temperature was kept at room temperature. The injection volume was 10 μL. The chromatographic column used for ZEA was Symmetry^®^ C18 with dimensions of 150 3.9 mm I.D and 5 μm particle size. The mobile phase was a 50% gradient of ACN in water eluted for 7 min. The injection volumes was 10 μL and the mobile phase flow rate was 1.0 mL/min. DON was determined using enzyme-linked immunosorbent assay (ELISA) described in Lee et al. [10].

### Conjugation of monoclonal antibodies (mAbs) and MNPs

A total of 2 mg (3 mg for DON) of MNP suspension was washed three times using a magnet in a coupling buffer (0.01 M pyridine, pH 6.0). A 5% aqueous glutaraldehyde solution (1 mL) was then added and reacted with MNPs at room temperature for 30 min in a shaking incubator (BioShaker M-BR-022UP, Taitec Corporation, Tokyo, Japan) (1,000 rpm). The particles were washed with coupling buffer by magnetic separation. Coupling with mAbs was achieved by dissolving 50 or 100 μg (kj-AFB, kk-ZEA) and 300 μg (NVRQS-DON) of mAbs in 500 μL of coupling buffer and then mixing the coupled solution with activated magnetic particles on a shaking incubator at 1,000 rpm at room temperature for 16 to 24 h. Following this, the coupling solution was quenched with 1 M glycine solution (pH 8.0) at room temperature for 30 min in a shaking incubator. MNPs coupled with the antibodies were washed and stored in the wash buffer at 4 °C until use.

### Purification of mycotoxins using mAb-coupled MNPs (mAb–MNPs) from buffer solution, swine feed, white soybeans and maize

To determine recovery rate of AFB_1_, ZEA, and/or DON, mAb–MNPs were mixed with 500 μL of buffer solution (16% MeOH in PBS) (AFB_1_: 5, 10 and 20 ng/mL; ZEA: 125, 250 and 500 ng/mL and DON: 250, 500 and 1,000 ng/mL) for 5 min at room temperature in a shaking incubator (1,000 rpm). Upon completion of the reaction, mAb–MNPs bound to each mycotoxin were magnetically separated from the supernatant, and the supernatant was carefully discarded. Each mycotoxin was detached from the complexes of mycotoxin and mAb–MNPs by the addition of 500 μL of 100% MeOH, with gentle shaking.

Swine feed, white soybeans, and maize were ground in a Waring blender (Model 51BL31) (Waring Products, Torrington, CT, USA) for 5 min at a high speed. The ground sample (5 g) was spiked with each mycotoxin alone or simultaneously at different concentrations (AFB_1_: 0, 5, 10 and 20 ng/g; ZEA: 0, 125, 250 and 500 ng/g and DON: 0, 250, 500 and 1,000 ng/g) and gently shaken by hand. The spiked samples were extracted by vigorous agitation with 25 mL of 70% MeOH in PBS for AFB_1_ and ZEA and 16% MeOH in PBS for DON. The extracts were filtered through Whatman No. 1 filter paper (110 mm diameter). The concentration of each mycotoxin in the extracted solution was determined using both ELISA and HPLC method after mAb–MNPs purification. In the case of purifying with mAb–MNPs, the extracted solution was diluted one-half with PBS because a high methanol concentration can damage the antibodies. The result was multiplied by the dilution factor. For the swine feed, white soybeans, and maize samples, mAb–MNPs were mixed with 500 μL of extracted sample containing 0, 5, 10 and 20 ng AFB_1_/g and/or 0, 125, 250 and 500 ng ZEA/g and/or 0, 250, 500 and 1,000 ng DON/g for 30 min at room temperature in a shaking incubator (1,000 rpm). Upon completion of the reaction, mAb–MNPs bound to each mycotoxin were magnetically separated from the supernatant, which was carefully discarded. Mycotoxins were dissociated from mAb–MNP complexes by the addition of 500 μL of 100% MeOH with gentle shaking. After dissociation, mAb–MNPs were magnetically separated perpendicular to gravity, and the supernatant was used to determine the quantity of each mycotoxin in the samples using the ELISA assay (DON) and HPLC method (AFB_1_ and ZEA) developed in our laboratory [10].

### *Purification of AFB*_*1*_ and ZEA from feed using an immunoaffinity column

For AFB_1_, ground feed samples (10 g) spiked with a known volume (at final concentrations of 5,10 and 20 ng/g) of an AFB_1_ stock solution were mixed with 20 mL of 80% MeOH/H_2_O (v/v) and 1 g of sodium chloride and blended at a high speed for 3 min to obtain a homogeneous sample mix. The mixture was centrifuged for 15 min at 1,600 g. Following this, 10 mL of an aqueous methyl alcohol phase was mixed with 40 mL of PBS solution. This diluted solution was filtered through a filter paper (Whatman No. 4, 55 mm diameter) and 20 mL was passed through an immunoaffinity column (Neogen, Glasgow, UK) at a flow rate of 1.5–2.0 mL/min. For further purification, 20 mL of 25% MeOH/H_2_O (v/v) was passed through the immunoaffinity column. AFB_1_ was then eluted from the column with 2 mL of HPLC-grade MeOH and then with 2 mL of HPLC-grade water using gravity to collect the eluate into a glass vial.

For ZEA, ground feed samples (5 g) spiked with a known volume (at final concentrations of 125, 250 and 500 ng/g) of an ZEA stock solution were mixed with 25 mL of 75% HPLC-grade ACN/H_2_O (v/v) using a high-speed mixer for 2 min. The mixture was centrifuged for 10 min at 1600×*g*. Following this, 20 mL of the aqueous ACN phase was mixed with 80 mL of PBS solution. After mixing, the diluted solution was filtered through a filter paper and 25 mL was transferred to an immunoaffinity column (R-BIOPHARM RHÔNE LTD, Glasgow, Scotland) at a flow rate of approximately 5 mL/min. For washing, 20 mL of PBS was passed through the immunoaffinity column. Bound ZEA was first eluted with 1.5 mL of HPLC-grade ACN and then with 1.5 mL of HPLC-grade water into the same vial.

## Results

MNPs were coupled with mAbs (kj-AFB, kk-ZEA and NVRQS-DON) against their specific mycotoxins: AFB_1_, ZEA and DON. The binding percentages of each mycotoxin antibody were high, ranging from 83.15 to 95.44% (Table 1). Purification of each of the three individual mycotoxins AFB_1_, ZEA and DON was performed using their specific mAb–MNPs. The recovery of each mycotoxin from the spiked buffer solution was confirmed by HPLC and ELISA. As described in Table 2, mean recovery values of AFB_1_ were 93.1–95.0% over concentrations ranging from 5 to 20 ng/g, with less than 0.86% coefficient of variation (CV). Recoveries of ZEA and DON from samples spiked with 125–500 ng/g of ZEA and 250–1000 ng/g of DON were 87.2–96.0% with less than 8.47% CV and 75.2–96.9% with less than 6.05% CV, respectively (Table 2).

**Table 1:** Binding capability of MNP to onoclonal antibodies of AFB_1_, ZEA and DON

Toxin type	Amount of MNP (mg)	Added amount of mAb (μg/ml)	Coupling amount of mAb (mean ± SD, µg/ml)	Binding capacity (mean ± SD, %)
AFB_1_	2	50	44.89 ± 6.46	83.15 ± 3.24
ZEA	2	100	94.64 ± 0.81	91.39 ± 1.14
DON	3	300	281.28 ± 3.30	95.44 ± 0.51

Each value represents the mean of seven replicate experiments (*n* = 7)

**Table 2:** Recovery of individual mycotoxins using MNP and specific mAb from spiked buffer solution

Toxin type	Spiked amount (ng/ml)	Measured (mean ± SD, ng/ml)	Recovery (mean ± SD, %)	Binding capacity (ng/µg)		CV (%)
AFB_1_	5	4.66 ± 0.04	93.1 ± 0.7	0.388 ± 0.003		0.86
	10	9.32 ± 0.05	93.2 ± 0.5		0.776 ± 0.004	0.54
	20	19.00 ± 0.13	95.0 ± 0.6		1.583 ± 0.010	0.68
ZEA	125	109.03 ± 9.24	87.2 ± 7.4		0.545 ± 0.046	8.47
	250	218.33 ± 1.89	87.3 ± 0.8		1.091 ± 0.009	0.87
	500	479.93 ± 15.08	96.0 ± 3.0		2.399 ± 0.075	3.14
DON	250	187.94 ± 8.27	75.2 ± 3.3		0.313 ± 0.013	4.40
	500	484.37 ± 29.31	96.9 ± 5.9		0.807 ± 0.048	6.05
	1000	880.88 ± 51.47	88.1 ± 5.2		1.468 ± 0.085	5.84

Each value represents the mean of three replicate experiments (*n* = 3)

The results of simultaneous purification of AFB_1_/ZEA, AFB_1_/DON and DON/ZEA using their specific mAb–MNPs are shown in Table 3. The analytical recoveries for 20 ng/g AFB_1_ and 500 ng/g ZEA directly spiked into the buffer solution were 87.0 and 99.8%, respectively. Recovery rates of AFB_1_/DON and DON/ZEA were 86.2%/76.6% and 92.0%/86.7%, respectively, at a concentration of 20 ng/g for AFB_1_, 500 ng/g for ZEA and 1000 ng/g for DON. We also attempted to simultaneously purify all three mycotoxins from spiked and mixed buffer solutions. Recoveries using the novel mAb–MNP conjugated system were 82.5, 94.6 and 73.4% in a buffer solution spiked with AFB_1_, ZEA and DON at concentrations of 20, 500 and 1000 ng/g, respectively (Table 4).

**Table 3:** Recovery of mycotoxins using MNP and specific mAb from buffer solution simultaneously spiked with two mycotoxins

Toxin type	Spiked amount (ng/ml)	Measured (mean ± SD, ng/ml)	Recovery (mean ± SD, %)	Binding capacity (ng/µg)	CV (%)
AFB_1_ + ZEA	20	17.40 ± 0.19	87.0 ± 0.9	1.449 ± 0.015	1.09
	500	498.86 ± 15.25	99.8 ± 3.1	2.494 ± 0.076	3.06
ZEA + DON	500	433.68 ± 41.14	86.7 ± 8.2	2.168 ± 0.205	9.49
	1000	920.39 ± 189.18	92.0 ± 18.9	1.534 ± 0.315	20.55
AFB_1_ + DON	20	17.25 ± 0.14	86.2 ± 0.7	1.437 ± 0.011	0.81
	1000	766.11 ± 36.88	76.6 ± 3.7	1.276 ± 0.061	4.81

Each value represents the mean of three replicate experiments (*n* = 3)

**Table 4:** Recovery of mycotoxins using MNP and specific mAb from buffer solution simultaneously spiked with three mycotoxins

Toxin type	Spiked amount (ng/ml)	Measured (mean ± SD, ng/ml)	Recovery (mean ± SD, %)	Binding capacity (ng/µg)	CV (%)
AFB_1_ + ZEA +DON	20	16.50 ± 0.40	82.5 ± 2.0	1.374 ± 0.033	2.42
	500	472.97 ± 23.02	94.6 ± 4.6	2.364 ± 0.115	4.87
	1000	734.33 ± 43.42	73.4 ± 4.3	1.223 ± 0.072	5.91

Each value represents the mean of three replicate experiments (*n* = 3)

The applicability of the novel mAb–MNP conjugated system for the purification of mycotoxins in cereals and swine feed samples was investigated in the present study. AFB_1_ and DON were selected for pre-experimental trials. Recoveries of AFB_1_ in animal feed were 81.8–110.1% at concentrations of 5–20 ng/g spiked in feed and those of DON were 107.7–132.5% at concentrations of 250–1000 ng/g. For maize, recoveries of AFB_1_and DON were 65.6–83.0% for AFB_1_ and 82.4–103.4% for DON. Low recoveries of AFB_1_ and DON were achieved for white soybean (Supplementary Table 1, 2). The IAC clean-up method was selected for comparison with the novel purification method using mAb–MNPs. After fortification of animal feed with AFB_1_ (5, 10 and 20 ng/g feed) and ZEA (125, 250 and 500 ng/g feed), AFB_1_ and ZEA were purified using both the methods. Mean recoveries for AFB_1_ were 89.4, 73.1 and 88.3%, at concentrations of 5, 10 and 20 ng/g, respectively. For ZEA, mean recoveries were 86.7, 85.9 and 79.1% at concentrations of 125, 250 and 500 ng/g, respectively. For IAC purification, recoveries were 42.9–45.1% for AFB_1_ and 96.8–103.2% for ZEA (Table 5).

**Table 5:** Recoveries of AFB_1_ and ZEA spiked in animal feed after MNP purification and IAC

Spiked amount(ng/g)	mAb–MNP purification		Immunoaffinity columns	
	Measured (mean ± SD, ng/g)	Recovery (mean ± SD, %)	Measured (mean ± SD, ng/g)	Recovery (mean ± SD, %)
AFB_1_ 5	4.47 ± 1.30	89.4 ± 26.0	2.17 ± 0.43	43.3 ± 8.7
10	7.31 ± 0.57	73.1 ± 5.7	4.29 ± 0.30	42.9 ± 3.0
20	17.66 ± 2.01	88.3 ± 10.1	9.01 ± 1.09	45.1 ± 5.5
ZEA 125	108.39 ± 5.67	86.7 ± 4.5	121.05 ± 10.15	96.8 ± 8.1
250	214.68 ± 13.20	85.9 ± 5.3	257.97 ± 50.47	103.2 ± 20.2
500	395.32 ± 35.27	79.1 ± 7.1	497.47 ± 30.23	99.5 ± 6.1

Each value represents the mean of three replicate experiments (*n* = 3)

## Discussion

Regulatory concentrations of AFB_1_, ZEA and DON have been established to reduce human health risk [12–14]. The extraction efficacy of any analytical method for mycotoxin testing is important for improving method accuracy. Although IAC is most commonly used to isolate mycotoxins from samples [3, 4], magnetic microbeads and nanoparticles have been suggested as alternative tools for the separation of chemicals in grains and biological samples [5, 6, 15]. Compared with microbead-based immunomagnetic separation, MNPs have the following advantages: good capture efficiency, no need for mechanical mixing, and minimal sample preparation. The poor solubility and extensive aggregation properties of MNPs are obstacles to their application to the separation of organic chemicals from samples. Quality and size control are important factors in recovery of mycotoxin from samples. Microsized beads have a low dispersion capacity in solution and can make the separation procedure laborious and the application of nanoparticles to the separation of chemicals from a biological sample requires a stable colloidal nanoparticle suspension because nanoparticles tend to agglomerate in a liquid solution. Comparatively, the MNPs used in the present study showed good dispersion. They were 100–150 nm in size and could be produced with a high yield and reproducibility between batches with a very homogenous particle size (data not shown). Their individual particle morphology is nearly spherical.

Although a broad surface area affords a greater opportunity for the binding of mAb, it is important that the Fab region be exposed because when the antibody binds to MNP because the Fab region is the site for antigen molecular recognition and binding. In this experiment, we first determined the ideal binding ratio of mAb to MNP and found that antibody coupled to MNPs exhibited a high binding capacity. We did not determine the type of binding of mAb to MNP, thus further modification may be required to increase the efficiency of binding between mAb and MNP [16].

Simultaneous purification of mycotoxins using mAb–MNP conjugates is attractive because it saves time and resources. In this experiment, recoveries of AFB_1_/ZEA, AFB_1_/DON and DON/ZEA spiked in the buffer solution were 87.0%/99.8%, 86.2%/76.6% and 92.0%/86.7%, respectively. Recoveries of AFB_1_, ZEA and DON spiked simultaneously were 82.5%, 94.6% and 73.4%, respectively. According to the Codex Alimentarius guidelines (CAC/GL 71-2009) for quantitative analytical methods, acceptable recovery ranges are 60%–120% with CV 30%, 70–120% with CV 20% and 70–110% with CV 15% for samples containing 1–10, 10–100 and 100–1000 ng/g of analyte, respectively. Recoveries from the buffer solution using mAb–MNP conjugates in the present study satisfied the Codex Alimentarius guidelines for the three types of mycotoxins. For the feed sample fortified with AFB_1_ and ZEA, mean recoveries for AFB_1_ and ZEA were 73.1–89.4% and 79.1–86.7%, respectively, both of which satisfied the standard. The present data indicate that our simultaneous purification experiments achieved high recovery of each mycotoxin, similar to the recovery rates obtained using the individual separation method.

We also applied this novel tool to cereals and medicinal herbal plants. The results showed that this novel tool could be used for the purification of DON and AFB_1_ in swine feed and maize, but low recovery rates were found in the case of white soybeans (supplementary Table 1, 2). Mycotoxins extracted from white soybeans were also determined by ELISA, which also showed a low recovery. In medicinal herbal plants, a dried root of Glycyrrhiza glabra (Liquorice, also Licorice) and seeds of Cassia tora, recoveries of DON were low (data not shown). The matrix is an important factor in the extraction of chemicals from vegetables and seafood [17]. We speculate that white soybeans and the plants used in herbal medicine contain some inhibitory component that reduces the binding of mAb–MNP conjugates to free mycotoxin in sample solutions. The present data indicate that the extraction efficiency depends on the matrix type and that a more advanced extraction method is required for mycotoxin isolation from white soybeans and some plants used in herbal medicine.

To determine the applicability of the novel system in feed or grain matrices, we compared our purification method using the novel mAb–MNP conjugated system with an IAC clean-up method. AFB_1_ and ZEA were extracted and purified from swine feed samples spiked with AFB_1_ and ZEA using both the methods. The mAb–MNP conjugated system showed a good recovery of both mycotoxins, whereas IAC showed a low recovery of AFB_1_. In contrast to IAC, the novel system can be simultaneously applied to separate several mycotoxins from feed or food. The present results showed that the mAb–MNP conjugated system could replace the IAC kit for the isolation of mycotoxins from some food matrices.

In conclusion, MNP–antibody conjugates used in the present study have the advantage that toxins and unbound materials can be separated by magnetism and that the washing process is simple and requires little extraction buffer. The purification method using mAb–MNPs can be used for simple and simultaneous purification of mycotoxins from feed and some grains.

## Supplementary Information

Below is the link to the electronic supplementary material.

Supplementary file1 (DOCX 16 KB)
