# Supplementary material for: Rapid and simultaneous purification of aflatoxin B1, zearalenone and deoxynivalenol using their monoclonal antibodies and magnetic nanoparticles
Source: Toxicol Res. 2021 Jan 28;37(4):421–7. doi: 10.1007/s43188-020-00083-w (PMC8476696; doi:10.1007/s43188-020-00083-w)
Supplement: Supplementary file 1 — Supplementary file1 (DOCX 16 KB) [file 43188_2020_83_MOESM1_ESM.docx]

Supplementary Table 1. Recoveries of AFB_1_ spiked in animal feed, white soybean and maize

after MNP purification

| Items | Spiked amount  (ng/g) | Measured  (ng/g) | Recovery  (%) | Binding capacity  (ng/µg) |
| --- | --- | --- | --- | --- |
| Feed | 5 | 5.51 | 110.1 | 0.459 |
|  | 10 | 8.18 | 81.8 | 0.682 |
|  | 20 | 18.18 | 90.9 | 1.515 |
| White soybeans | 5 | 0.00 | 0.00 | 0.000 |
|  | 10 | 4.40 | 44.0 | 0.366 |
|  | 20 | 10.50 | 52.5 | 0.875 |
| Maize | 5 | 3.60 | 71.9 | 0.300 |
|  | 10 | 8.30 | 83.0 | 0.692 |
|  | 20 | 13.11 | 65.6 | 1.093 |

  Supplementary Table 2. Recoveries of DON spiked in animal feed, white soybean and maize

after MNP purification

| Items | Spiked amount  (ng/g) | Measured  (ng/g) | Recovery  (%) | Binding capacity  (ng/µg) |
| --- | --- | --- | --- | --- |
| Feed | 250 | 310.70 | 124.3 | 0.518 |
|  | 500 | 662.50 | 132.5 | 1.104 |
|  | 1000 | 1076.90 | 107.7 | 1.795 |
| White soybeans | 250 | 83.10 | 33.2 | 0.139 |
|  | 500 | 281.10 | 56.2 | 0.469 |
|  | 1000 | 449.10 | 44.9 | 0.749 |
| Maize | 250 | 206.00 | 82.4 | 0.343 |
|  | 500 | 509.40 | 101.9 | 0.849 |
|  | 1000 | 1033.60 | 103.4 | 1.723 |
